# Analysis of complete mitogenomes and phylogenetic relationships of *Frontopsylla spadix* and *Neopsylla specialis*

**DOI:** 10.3389/fvets.2023.1250381

**Published:** 2023-09-07

**Authors:** Yafang Liu, Bin Chen, Xinyan Lu, Shuang Liu, Dandan Jiang, Xuan Wang, Lin Yi, Rongyu Li, Quanfu Zhang, Lixian Wu, Xing Yang

**Affiliations:** ^1^Integrated Laboratory of Pathogenic Biology, College of Preclinical Medicine, Dali University, Dali, China; ^2^School of Public Health, Dali University, Dali, China; ^3^Queen Mary School, Nanchang University, Nanchang, China; ^4^College of Preclinical Medicine, Dali University, Dali, China; ^5^Department of Gastroenterology, Clinical Medical College and the First Affiliated Hospital of Chengdu Medical College, Sichuan, China

**Keywords:** *Frontopsylla spadix*, *Neopsylla specialis*, flea, mitochondrial genome, phylogenetic

## Abstract

Fleas represent a group of paramount medical significance, subsisting on blood and acting as vectors for an array of naturally occurring diseases. These pathogens constitute essential elements within the plague biome, exerting deleterious effects on both human and livestock health. In this study, we successfully assembled and sequenced the whole mitochondrial genome of *Frontopsylla spadix* and *Neopsylla specialis* using long-range PCR and next-generation sequencing technologies. The mitogenomes of *F. spadix* and *N. specialis* both have 37 genes with full lengths of 15,085 bp and 16,820 bp, respectively. The topology of the phylogenetic tree elucidates that species *F. spadix* is clustered in a branch alongside other members of the family Leptopsyllidae, whereas species *N. specialis* is a sister taxon to *Dorcadia ioffi* and *Hystrichopsylla weida qinlingensis*. It also suggests that Pulicidae form a monophyletic clade, Ctenopthalmidae, Hystrichopsyllidae, Vermipsyllidae form a sister group to Ceratophyllidae/Leptopsyllidae group. The mitochondrial genomes of *F. spadix* and *N. specialis* were sequenced for the first time, which will contribute to a more comprehensive phylogenetic analysis of the Siphonaptera order. The foundation for subsequent systematic studies, and molecular biology of fleas was established.

## Introduction

Fleas (Order Siphonaptera) are small, wingless insects with laterally compressed bodies undergo holometaboliam and parasitize mammals and birds (1, 2). Over, 2500 species of fleas have been identified, with approximately 200 species capable of harboring epidemic bacteria (3, 4). These serve as vectors for a range of pathogens, including Rickettsia, Bartonella, bubonic plague, and Tularemia, thereby functioning as both disease-causing vectors and reservoir hosts (5, 6). As a result of changes in the environment and human behavior, the vector-host ecology has changed, increasing human exposure to flea vectors and the pathogens they transmit, and flea-borne diseases may re-emerge as epidemics (7). Fleas are an early warning indicator of the plague epidemic, which has important significance in medicine and veterinary medicine (8). Fleas and flea-borne diseases are increasingly threatening human and animal health and causing serious economic losses, so flea identification is of great practical importance for flea-borne disease prevention and control.

Both *Frontopsylla spadix* and *Neopsylla specialis* are found in the Siphonaptera order, belonging to the families Leptopsyllidae and Ctenophthalmidae, to the Amphipsyllinae and Neopsyllinae, and *Frontopsylla* and *Neopsylla*, respectively. Species *F. spadix* are parasitic on wild rodents, specifically *Apodemus chevrieri* and *Rattus flavipectus*, found in regions such as Yunnan, Gansu, and Tibet in China, and extending into Nepal. This geographic distribution is considered a conduit for the transference of plague from wild to domestic rodents (9). Species *N. specialis*, parasitizing mammals like *Apodemus chevrieri, Apodemus draco*, and *Apodemus latronum*, is in China and acts as a principal vector of the plague in Yunnan's natural foci, akin to species *F. spadix* (10). Morphological characteristics of *F. spadix* and *N. specialis* have been described previously, the identification resolution of traditional flea classification methods is low, which may have certain limitations (2).

Mitochondria are placed in cells that produce energy and have a separate set of genetic material called mitochondrial DNA (11). Mitochondrial DNA is one of the most commonly used molecular markers in systematics and is widely used in phylogenetic studies of different organisms because of its simple structure, maternal inheritance, and rapid evolutionary rate (12). The analysis of mitochondrial genome structure and sequence is helpful to clarify the classification, genetic evolution, and phylogenetic relationship of fleas more clearly (13). However, at present, the mitochondrial genome data of fleas is very limited, resulting in a huge obstacle to fleas and flea-borne diseases. Therefore, we need to continuously increase and improve the flea mitochondrial gene database to lay the foundation for flea taxonomy, population genetics, and phylogeny.

In this study, we provide the first complete description of the mitochondrial genomes of *F. spadix* and *N. specialis*, analyze mitogenome structures to address the lack of mitochondrial gene resources in fleas, and construct the phylogenetic relationships of known mitochondrial genomes in the order Siphonaptera, while providing molecular information for flea prevention and control.

## Materials and methods

### Sample collection and DNA extraction

Adult specimens of *F. spadix* (one female and one male) were collected in July 2020 from Luoping Mountain, eryuan City, Dali Bai Autonomous Prefecture, Yunnan Province, China (26°07′N, 99°85′E). Three females and one male adult specimens of *N. specialis* were found in June 2022 from Laojun Mountain, Lijiang City, Yunnan Province of China (26°53′N, 99°58′E). Species identification was conducted based on morphological characteristics with *F. spadix* and *N. specialis* samples extracted from an adult female using the TIANamp Genomic DNA Kit (TIANGEN, Beijing, China) following the manufacturer's instructions.

### PCR amplification

The study design included the development of two sets of overlapping long fragment PCR primers to amplify the mitochondrial genomes of *F. spadix* and *N. specialis*. This was conducted using *cox1* and *12S rRNA* genes of *Ctenophthalmus quadratus* (OQ023577) and *Leptopsylla segnis* (OQ023576), with primer design achieved through Primer 5.0 software, as delineated in Table 1. The PCR was performed in the 50 μl system, including 10 μl 5× PrimerSTAR GXL Buffer (Takara, Japan), 4 μl of each primer, 4 μl of dNTPs, 1 μl of PrimerSTAR GXL DNA Polymerase (Takara, Japan), 4 μl of DNA template and 23 μl of ddH_2_O under the following reaction conditions: 92°C for 2 min for initial denaturation, followed by 35 cycles of denaturation at 92°C for 10 s, annealing at 68°C for 30 s and extension at 68°C for 10 min and the final 68°C extension time of 10 min. PCR amplification products were detected by electrophoresis on 1% agarose gels, purified, and sequenced by Sangon Biotech Company (Shanghai, China).

**Table 1:** PCR primers for sequencing the mitogenome of *F. spadix* and *N. specialis*.

**Primes**	**Sequence (5^′^-3^′^)**
FS1F	ATAGGAGCAGTATTCGCAATTATAGCC
FS1R	ACTATCAGGATAATCAGAGTAACGTCG
FS2F	CGTGGATTATCGATTACAGAACAGG
FS2R	GCAGCTGCGGTTATACAATTAA
NS1F	TGATTAGCAACTCTACACGGAAGAA
NS1R	AATGGAAATCAGTGAACGAATCCTG
NS2F	CCTTCCGGTACACCTACTTTGTTA
NS2R	CAAGGTGCAGTTAATGGTTTAGTAG

### Gene annotation

Sequencing employed next-generation sequencing technology (NGS) on the Illumina NovaSeq platform. Annotations were facilitated via the MITOS WebServer (http://mitos.bioinf.uni-leipzig.de/index.py), with the A5-miseq v20150522 program utilized for the construction of the complete mitochondrial genome (14, 15). Alignment with closely related species in the NCBI database were performed to ascertain the location of protein-coding, tRNA, and rRNA genes. Predictive analyses of tRNA genes secondary structure were conducted on the tRNAscan-SE (http://lowelab.ucsc.edu/tRNAscan-SE/) online platform (16), and the CGView Server (https://paulstothard.github.io/cgview/) was employed for mapping mitochondrial genome circles. Analytical tools included DNAStar V7.1 for nucleotide composition analysis and CodonW 1.4.2 the relative synonymous codon usage (RSCU) computation.

### Phylogenetic analysis

The 13 protein-coding gene sequences from 15 flea species were independently aligned using MUSCLE nucleotide mode, and datasets were manually concatenated. Positions containing gaps and incomplete data were excluded through Bioedit v7.0.5.3 software. Phylogenetic relationships were analyzed using *Casmara patrona* as an outgroup (Table 2), and trees were constructed with MEGA 7.0 software and Mrbayes v.3.2.7 software. The ML tree was formulated using GTR+G+I as the optimal model based on the Akaike Information Criterion (AIC) (17), employing the maximum likelihood method across 1,000 bootstrap datasets. The BI tree underwent 10,000,000 generations, sampled every 1,000 generations. The evolutionary relationships among flea species were visually depicted using the software Figtree v1.4.2.

**Table 2:** Mitochondrial genome sequence information used in this paper.

**Species**	**Family**	**Length (bp)**	**Accession number**
*Ceratophyllus anisus*	Ceratophyllidae	15,875	OQ366407.1
*Ceratophyllus wui*	Ceratophyllidae	18,081	NC040301.1
*Paradoxopsyllus castodis*	Leptopsyllidae	15,375	OQ627398.1
*Jellisonia amadoi*	Ceratophyllidae	17,031	NC022710.1
*Leptopsylla segnis*	Leptopsyllidae	15,785	OQ023576.1
*Frontopsylla spadix*	Leptopsyllidae	15,085	OQ366408.1
*Neopsylla specialis*	Ctenophthalmidae	16,820	OQ366409.1
*Hystrichopsylla weida qinlingensis*	Hystrichopsyllidae	17,173	NC042380.1
*Dorcadia ioffi*	Vermipsyllidae	16,785	NC036066.1
*Pulex irritans*	Pulicidae	20,337	NC063709.1
*Xenopsylla cheopis*	Pulicidae	18,902	MW310242.1
*Ctenocephalides canis*	Pulicidae	15,609	ON109770.1
*Ctenocephalides orienties*	Pulicidae	22,189	NC073009.1
*Ctenocephalides felis*	Pulicidae	15,418	MK941844.1
*Ctenocephalides felis*	Pulicidae	20,873	MT594468.1
*Casmara patrona*	Oecophoridae	15,393	NC053695.1

## Results

### Organization of mitochondrial genome

**Figure 1:**
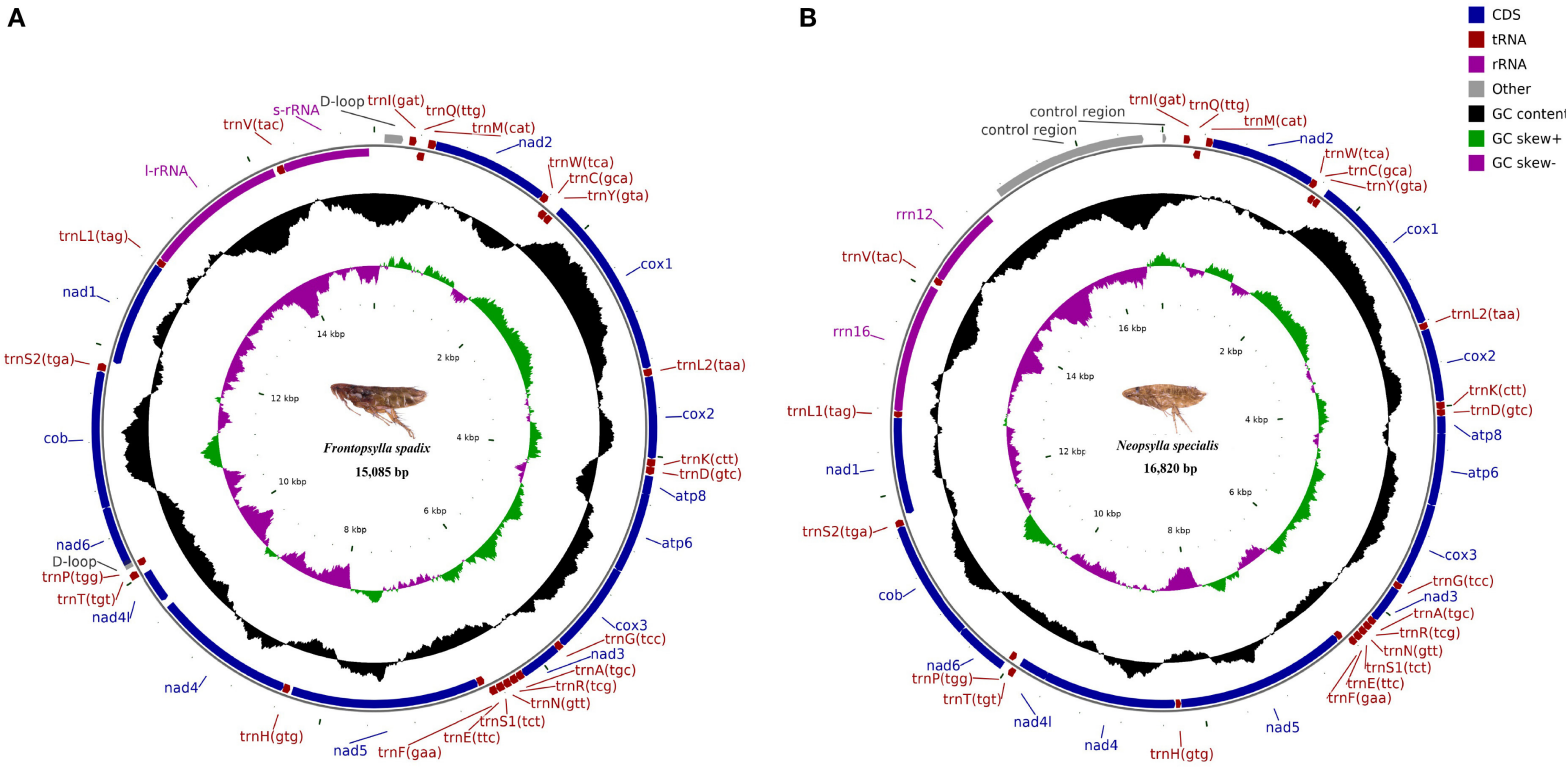
Map of the mitochondrial genome of *Frontopsylla spadix* **(A)** and *Neopsylla specialis* **(B)**. Genes in the inner circle plot are located on the reverse strand, and the rest of the genes are located on the forward strand.

The mitochondrial genomes of *F. spadix* and *N. specialis*, which are typically closed double-stranded molecular structures, were uploaded to Genbank in TBL format and obtained accession numbers OQ366408 and OQ366409, respectively. The length of the mitochondrial genome was 15,085 bp and 16,820 bp, respectively (Figure 1), with differences in length mainly determined by the length of the control region. Most of the genes including 14 tRNAs and 9 PCGs are distributed on the positive strand, the same as the other fleas (18, 19). Both intergenic regions and overlapping domains are present within the mitochondrial genome (Table 3). The mitogenomes of *F. spadix* and *N. specialis* had a significant AT preference with AT content of 78.83% and 77.27%, respectively, and the base content was 37.99% (38.64%) A, 40.84% (38.63%) T, 12.85 (14.22%) C, and 8.31 (8.51%) G (Table 4).

**Table 3:** Summary of the mitogenome of *F. spadix* and *N. specialis*.

**Gene**	**Strand**	**Position**	**Size(bp)**	**Initiation codon**	**Stop codon**	**Anticodon**	**Intergenic nucleotide**
D-loop	N	0/1-36	0/36				303/35
trnI	N	304-366/205-267	63/63			GAT	16/42
trnQ	J	451-383/378-310	69/69			TTG	17/43
trnM	N	469-536/422-487	68/66			CAT	
nad2	N	537-1547/488-1498	1011/1011	ATT/ATT	TAA/ TAA		−2/−2
trnW	N	1546-1610/1497-1560	65/64			TCA	−1/−8
trnC	J	1676-1610/1613-1553	67/61			GCA	
trnY	J	1739-1677/1676-1614	63/63			GTA	−3/−3
cox1	N	1737-3272/1674-3209	1536/1536	ATC/ATC	TAA/ TAA		4/4
trnL2	N	3277-3340/3214-3277	64/64			TAA	1/1
cox2	N	3342-4022/3279-3959	681/681	ATG/ATG	TAA/ TAG		2/2
trnK	N	4025-4094/3962-4031	70/70			CTT	−1/−1
trnD	N	4094-4159/4031-4093	66/63			GTC	9/0
atp8	N	4169-4330/4094-4261	162/168	ATA/ATT	TAA/ TAA		−7/−7
atp6	N	4324-4998/4255-4926	675/672	ATG/ATG	TAA/ TAA		−1/−1
cox3	N	4998-5780/4926-5708	783/783	ATG/ATG	TAA/ TAA		
trnG	N	5781-5842/5709-5770	63/62			TCC	
nad3	N	5843-6193/5771-6121	351/351	ATT/ATT	TAG/ TAG		−2/−2
trnA	N	6192-6256/6120-6182	65/63			TGC	−2/0
trnR	N	6255-6318/6183-6245	64/63			TCG	
trnN	N	6319-6383/6246-6309	65/64			GTT	
trnS1	N	6384-6452/6310-6378	69/69			TCT	
trnE	N	6453-6518/6379-6442	66/64			TTC	−2/−2
trnF	J	6581-6517/6504-6441	65/64			GAA	0/−1
nad5	J	8315-6582/8221-6504	1734/1718	ATG/ATG	TAA/TA		1/1
trnH	J	8381-8317/8276-8223	65/54			GTG	−1/−5
nad4	J	9717-8381/9623-8282	1337/1342	ATG/ATG	TTA/T		−7/−7
nad4l	J	10004-9711/9910-9617	294/294	ATG/ATG	TAA/ TAA		2/2
trnT	N	10007-10071/9913-9977	65/65			TGT	
trnP	J	10134-10072/10040-9978	63/63			TGG	11/17
nad6	N	10146-10652/10049-10558	507/510	ATA/ATT	TAA/ TAA		−1/−1
cob	N	10652-11791/10558-11691	1140/1134	ATG/ATG	TAA/ TAA		3/2
trnS2	N	11795-11860/11693-11757	66/65			TGA	20/18
nad1	J	12813-11881/12717-11776	933/942	ATG/ATG	TAA/ TAA		1/1
trnL1	J	12876-12815/12780-12719	62/62			TAG	
rrnL	J	14157-12877/14042-12781	1281/1262				28/33
trnV	J	14252-14186/14142-14076	67/67			TAC	−1/−1
rrnS	J	15037-14252/14932-14142	786/791				47/206
D-loop	N	0/15139-16638	0/1500				0/181

**Table 4:** Composition and skewness of *F. spadix* and *N. specialis* mitogenome.

**Region**	**A%**	**C%**	**G%**	**T%**	**A+T%**	**G+C%**	**AT Skew**	**GC Skew**
Whole genome	37.99/38.64	12.85/14.22	8.31/8.51	40.84/38.63	78.83/77.27	21.16/22.73	−0.036/0.001	−0.215/−0.251
nad2	35.41/34.32	10.68/13.45	7.12/7.81	46.79/44.41	82.20/78.73	17.80/21.27	−0.138/−0.128	−0.200/−0.265
cox1	29.43/27.67	15.76/17.77	14.13/14.78	40.69/39.78	70.12/67.45	29.88/32.55	−0.161/−0.180	−0.055/−0.092
cox2	35.24/33.04	13.51/17.18	9.99/11.01	41.26/38.77	76.51/71.81	23.49/28.19	−0.079/−0.080	−0.150/−0.219
atp8	42.59/41.07	5.56/10.12	3.09/5.36	48.77/43.45	91.36/84.52	8.64/15.48	−0.068/−0.028	−0.286/−0.307
atp6	33.04/32.14	13.33/16.07	9.19/10.12	44.44/41.67	77.48/73.81	22.52/26.19	−0.147/−0.129	−0.184/−0.227
cox3	30.65/29.76	14.69/16.09	12.52/13.41	42.15/40.74	72.80/70.50	27.20/29.50	−0.158/−0.156	−0.080/−0.091
nad3	29.06/29.63	13.68/15.67	7.69/8.26	49.57/46.44	78.63/76.07	21.37/23.93	−0.261/−0.221	−0.280/−0.310
nad5	36.85/33.41	7.38/7.63	12.40/14.73	43.37/44.24	80.22/77.65	19.78/22.35	−0.081/−0.139	0.254/0.318
nad4	34.93/31.22	7.18/8.20	13.31/14.98	44.50/45.60	79.43/76.83	20.49/23.17	−0.120/−0.187	0.299/0.293
nad4l	38.78/31.97	3.06/5.44	12.24/12.93	45.92/49.66	84.69/81.63	15.31/18.37	−0.084/−0.217	0.600/0.408
nad6	34.91/37.45	10.06/11.37	5.52/6.86	49.51/44.31	84.42/81.76	15.58/18.24	−0.173/−0.084	−0.291/−0.248
cob	31.67/30.16	16.14/17.02	10.88/11.02	41.32/41.80	72.98/71.96	27.02/28.04	−0.132/−0.162	−0.195/−0.214
nad1	31.94/30.89	7.18/7.43	15.11/15.82	45.77/45.86	77.71/76.75	22.29/23.25	−0.180/−0.195	0.356/0.361
rrnl	43.17/39.14	5.31/6.26	11.71/13.31	39.81/41.28	82.98/80.43	17.02/19.57	0.040/−0.027	0.376/0.360
rrns	40.84/39.32	6.62/6.95	11.96/14.03	40.59/39.70	81.42/79.01	18.58/20.99	0.003/−0.005	0.287/0.337
trnI	39.68/38.10	7.94/7.94	12.70/12.70	39.68/41.27	79.37/79.37	20.63/20.63	0/−0.040	0.231/0.231
trnQ	40.58/37.68	4.35/4.35	11.59/13.04	43.48/44.93	84.06/82.61	15.94/17.39	−0.034/−0.088	0.454/0.500
trnM	38.24/36.36	19.12/19.70	10.29/10.61	32.35/33.33	70.59/69.70	29.41/30.30	0.083/0.239	−0.300/−0.300
trnW	44.62/42.19	12.31/10.94	7.69/9.38	35.38/37.50	80.00/79.69	20.00/20.31	0.116/0.059	−0.231/−0.077
trnC	49.25/39.34	5.97/9.84	10.45/16.39	34.33/34.33	83.58/73.77	16.42/26.23	0.179/0.067	0.277/0.250
trnY	39.68/41.27	9.52/6.35	15.87/15.87	34.92/36.51	74.60/77.78	25.40/22.22	0.064/0.061	0.250/0.428
trnL2	29.69/35.94	15.62/15.62	14.06/14.06	40.62/34.38	70.31/70.31	29.69/29.69	−0.155/0.022	−0.053/−0.053
trnK	34.29/35.71	15.71/15.71	15.71/15.71	34.29/32.86	68.58/68.58	31.42/31.42	0/0.042	0/0
trnD	46.97/41.27	6.06/9.52	7.58/11.11	39.39/38.10	86.36/79.37	13.64/20.63	0.088/0.040	0.111/0.077
trnG	40.32/41.94	8.06/9.68	9.68/9.68	41.94/38.71	82.26/80.65	17.74/19.35	−0.020/0.040	0.091/0
trnA	38.46/42.86	7.69/6.35	9.23/11.11	44.62/39.68	83.08/82.54	16.92/17.46	−0.074/0.039	0.091/0.273
trnR	39.06/39.68	14.06/11.11	9.38/9.52	37.50/39.68	76.56/79.37	23.44/20.63	0.001/0	−0.200/−0.077
trnN	44.62/48.44	7.69/10.94	9.23/10.94	38.46/29.69	83.08/78.12	16.92/21.88	0.074/0.240	0.091/0
trnS1	39.13/39.13	10.14/10.14	10.14/10.14	40.58/40.58	79.71/79.71	20.29/20.29	−0.018/−0.018	0/0
trnE	42.42/42.19	6.06/6.25	4.55/4.69	46.97/46.88	89.39/89.06	10.61/10.94	−0.051/−0.053	−0.142/−0.143
trnF	40.00/34.38	7.69/9.38	15.38/15.62	36.92/40.62	76.92/75.00	23.08/25.00	0.040/−0.083	0.333/0.250
trnH	40.00/35.19	3.08/3.70	13.85/18.52	43.08/42.59	83.08/77.78	16.92/22.22	−0.037/−0.095	0.637/0.667
trnT	40.00/40.00	7.69/7.69	9.23/9.23	43.08/43.08	83.08/83.08	16.92/16.92	−0.037/−0.037	0.091/0.091
trnP	39.68/38.10	4.76/4.76	14.29/15.87	41.27/41.27	80.95/79.37	19.05/20.63	−0.020/−0.040	0.500/0.539
trnS2	42.42/43.08	6.06/6.15	12.12/12.31	39.39/38.46	81.82/81.54	18.18/18.46	0.037/0.057	0.333/0.334
trnL1	40.32/38.71	6.45/6.45	12.90/12.90	40.32/41.94	80.65/80.65	19.35/19.35	0/−0.040	0.333/0.333
trnV	43.28/44.78	7.46/7.46	5.97/5.97	43.28/41.79	86.57/86.57	13.43/13.43	0/0.035	−0.111/−0.111
OH	0/45.31	0/9.38	0/3.78	0/41.54	0/86.85	0/13.15	0/0.043	0/−0.426

### Protein-coding genes

**Figure 2:**
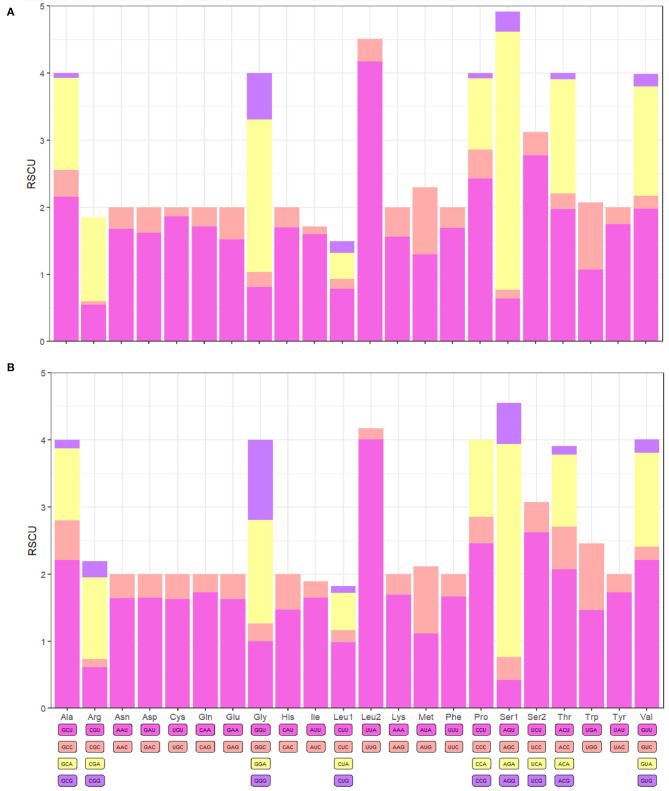
*F. spadix* **(A)** and *N. specialis* **(B)** protein-coding gene relative synonymous codon usage (RSCU).

The PCGs of *F. spadix* and *N. specialis* were 11,144 bp and 11,142 bp long, accounting for 73.87% and 66.24% of the complete mitochondrial genome length, respectively. Of the 13 protein-coding genes (PCGs) of *F. spadix*, which encode a total of 3713 codons, the initiation codon is the standard codon ATN, with TAA as the termination codon except for *NAD3* (TAG). *N. specialis* encodes a total of 3714 codons, with incomplete termination codons occurring in *NAD5* and *NAD4*, and *NAD3* with TAG as a stop codon. Leucine is the dominant amino acid and cysteine is the rarest amino acid (Figure 2). The mitochondrial genomes of *F. spadix* and *N. specialis* are mostly nonpolar amino acid groups with 1851 (49.85%) and 1933 (52.05%), respectively, and the remaining polar, basic, and acidic amino acid groups are 1209 (32.51%) and 1206 (32.47%), 235 (6.33%) and 230 (6.19%), 194 (5.22%) and 157 (4.23%).

### Transfer RNA genes and ribosomal RNA genes

The mitogenomes of *F. spadix* and *N. specialis* have 14 tRNAs located in the positive strand with full lengths of 1439 bp and 1408 bp, respectively. Among the 22 tRNA genes of *F. spadix*, the length of tRNA genes ranged from 62 bp (trnL1) to 70 bp (trnK), with the shortest amino acid of 61 bp (trnC) in *N. specialis*. The mitochondrial genome was conjured according to the special genetic code so that all the 22 tRNA genes could be identified. G-U oscillating bases appear as a common mismatch in most tRNA genes to maintain tRNA secondary structure (20). The relationship between base mismatch and evolution needs further consideration. With a length of 7 bp, which is typical of arthropods, *ATP8* and *ATP6* overlap (21). The *16S rRNA* and *12S rRNA* of *F. spadix* and *N. specialis* are both located in the reverse strand, separated by *Valine*, with AT contents of 82.98% (80.43%) and 81.42% (79.01%), respectively (Table 4).

### Phylogenetic analysis

**Figure 3:**
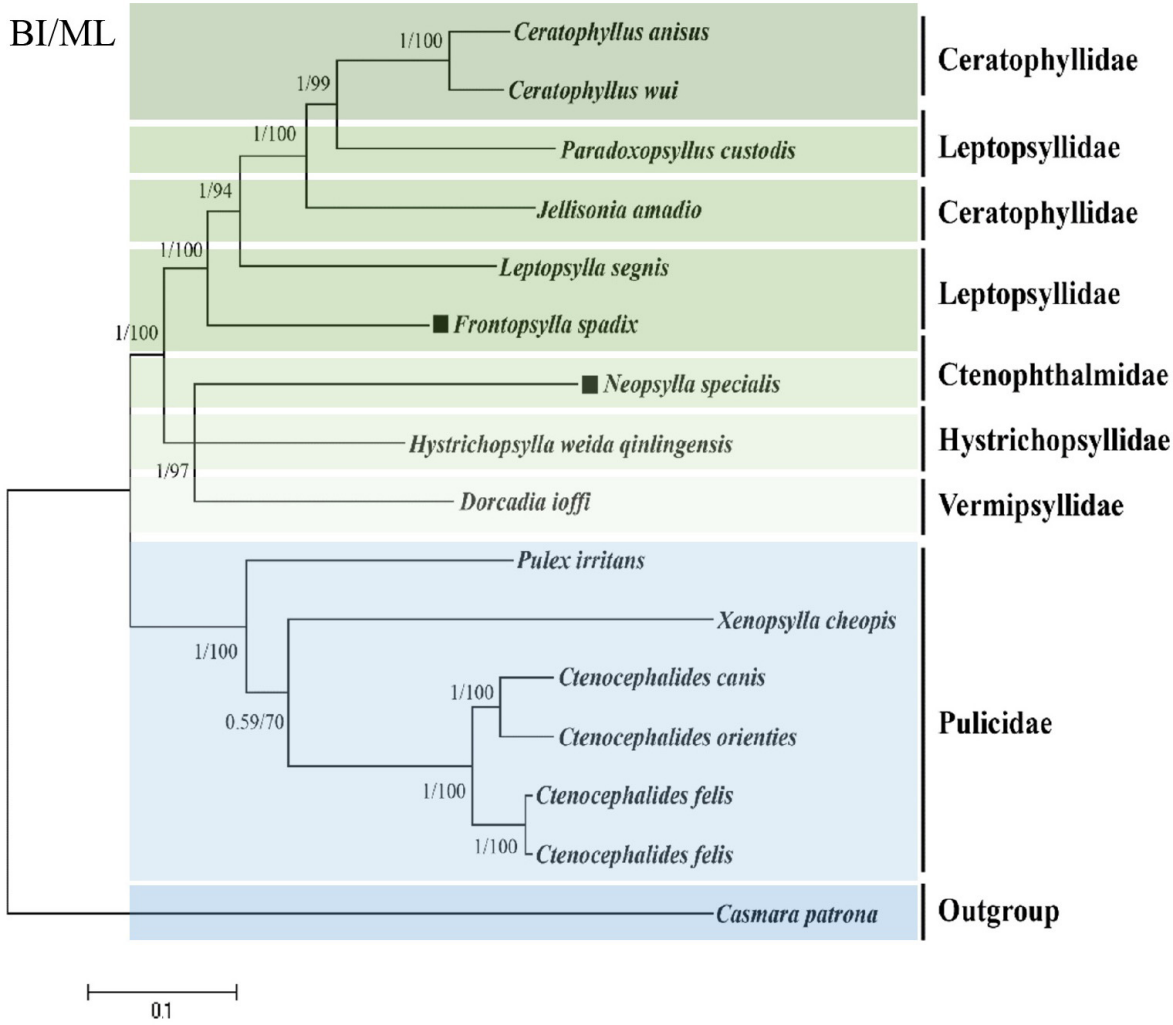
Phylogenetic analysis based on the nucleotide sequences of the 13 PCGs in the mitogenome. The number beside the nodes are posterior probabilities (BI) and bootstrap (ML). The black square markers represent the species in this study.

We harnessed available flea genomic data from the NCBI database pertaining to fleas and amalgamated this with our successfully sequenced *F. spadix* and *N. specialis*, thus facilitating a refined exploration of the topology of flea phylogenetic relationships. Utilizing the maximum likelihood method, we constructed a phylogenetic tree anchored on the concatenated nucleotide sequences of 13 PCG genes, thereby providing an insightful perspective into flea evolutionary trajectories. The ML and BI trees show identical topologies. According to ML and BI analysis, the families Ctenopthalmidae, Hystrichopsyllidae, Vermipsyllidae, and Pulicidae form a monophyletic clade, while the family Ceratophyllidae and Leptopsyllidae are paraphyletic. A principal clade encompasses species of the family Pulicidae, crystallizing into a definitive monophyletic clade. In juxtaposition, Ctenopthalmidae, Hystrichopsyllidae, and Vermipsyllidae form a sister aggregation to the Ceratophyllidae and Leptopsyllidae group. Notably, *F. spadix* and *Leptopsylla segnis* belonging to the Leptopsyllidae family emerge as the most phylogenetically congruent entities, bolstered by robust node support values. *N. specialis* resides solitarily on a branch, constituting a strongly endorsed linkage with *Dorcadia ioffi* and *Hystrichopsylla weida qinlingensis* and fostering sister group affiliations (Figure 3).

## Discussion

The endeavor to taxonomically identify and comprehend the ecological proclivities of fleas stands as an integral foundation in the mitigation and management of a plethora of naturally transpiring diseases. Fleas, being a medically salient insect group, partake substantially in perpetuating major plague epidemics and preserving natural epidemiological origin (22). Their abundance and widespread dissemination render them instrumental in the animal-mediated propagation of diseases during epidemic occurrences. Species *F. spadix* and *N. specialis*, as principal flea vectors, are posited to serve as conduits for the transmission of wild rodent plagues into domestic rats, thereby instigating epidemics.

In an examination of mitogenomes of *F. spadix* and *N. specialis* a pronounced predilection for AT bases was discerned, with an AT content surpassing that of GC, a feature congruent with arthropods (23). An anomalous stop codon was detected in *N. specialis*, subsequently rectified to TAA by PolyA complementation to terminate translation (24). The control region, harboring initiation sites that govern the replication and transcription of the mitochondrial genome, evolves at a rate three to five times that of other regions, accounting for its employment in population genetics and origin evolution studies (25, 26). Variability in the number and location of non-coding regions is evidenced across species, with singular, dual, and triple D-Loops manifesting in different species. Within this study's purview, species *F. spadix* was devoid of D-Loop, while species *N. specialis* harbored two, spanning 1,536 base pairs.

The six families are split into two large clades, as shown by phylogenetic clustering, except the family Pulicidae, where the remaining five families cluster in the other clade, with Paradoxopsyllus custodis located in the Superfamily Ceratophylloidae as a member of the family Leptopsyllidae, which is a discovery that also indicates that expanded sequencing of mitochondrial genomic data is beneficial for more intensive phylogenetic studies of the species. However, individual mitochondrial genes are less informative than the whole mitogenome which may bias the reflection of phylogenetic relationships (27), and in order to make the phylogenetic relationships of the flea more convincing, we need to sequence the whole mitochondrial genome of the flea more frequently (11).

Accurate differentiation and identification of flea species are essential in the diagnosis of disease and basic and applied research on these important ectoparasites. The mitochondrial genome is frequently used in phylogenetic and phylogenetic studies of different ectoparasites at various taxonomic levels due to its matrilineal inheritance, lack of recombination, and rapid rate of evolution. The in-depth analysis of the mitochondrial genomes of *F. spadix* and *N. specialis* augments the data corpus, fortifying further phylogenetic inquiry within the Siphonaptera order. This enhances both the resolution at the family echelon and the informativeness of the phylogenetic tree. The entire mitochondrial genome sequence has also been demonstrated to proffer elevated phylogenetic precision, rendering it an apt molecular marker for elucidating the evolutionary interconnections amongst flea species. Nevertheless, the sequencing of additional flea mitochondrial genomes is requisite to facilitate a more systematized and encompassing analysis of flea evolutionary relationships.

## Conclusion

In this study, the mitochondrial genomes of *F. spadix* and *N. specialis* were successfully sequenced based on the combination of long-range PCR technology and next-generation sequencing technology. In both *F. spadix* and *N. specialis*, the mitochondrial genomes are circular with the same genetic composition and arrangement as other fleas, which provides the basis for further understanding of the molecular evolution, and phylogeny of fleas, as well as providing useful molecular markers for studying the taxonomy and systematics of the flea species.

## Data availability statement

The datasets presented in this study can be found in online repositories. The names of the repository/repositories and accession number(s) can be found in the article/supplementary material.

## Ethics statement

The animal study was approved the Laboratory Animal Management Committee of Dali University and First Affiliated Hospital of Chengdu Medical College. The study was conducted in accordance with the local legislation and institutional requirements.

## Author contributions

YL conceived the study and wrote the manuscript. BC, XL, SL, and DJ collected specimens and participated in experimental operations. XW, LY, and RL analyzed the experimental data. QZ, LW, and XY are responsible for the interpretation of experimental data, critical revision of important knowledge content, and final approval of the version to be published. All authors contributed to the article and approved the submitted version.
